# *catena*-Poly[[diaquazinc(II)]-μ_3_-2,2′-dihydr­oxy-1,1′-binaphthyl-3,3′-di­carboxyl­ato-κ^3^ *O*:*O*′:*O*′′]

**DOI:** 10.1107/S1600536809051836

**Published:** 2009-12-09

**Authors:** Ling-Yun Zhang

**Affiliations:** a Department of Technology, Guangdong Police Officers College, Guangzhou, Guangdong 510230, People’s Republic of China

## Abstract

In the title coordination complex, [Zn(C_22_H_12_O_6_)(H_2_O)_2_]_*n*_ or [Zn(H_2_nba)(H_2_O)_2_]_*n*_ (H_2_nba is 2,2′-dihydr­oxy-1,1′-bi­naph­thyl-3,3′-dicarboxyl­ate), the Zn^II^ atom is coordinated by three H_2_nba ligands and two water molecules, resulting in a distorted trigonal-bipyramidal geometry. In the crystal structure, adjacent Zn^II^ atoms are linked by two H_2_nba ligands, forming one-dimensional ribbons along the *c* axis. These ribbons are further assembled into layers parallel to the *bc* plane *via* O—H⋯O hydrogen bonds.

## Related literature

For *d* ^10^ metal complexes with the H_2_nba ligand, see: Han *et al.* (2008 ▶); Zheng *et al.* (2004 ▶). For the potential coordination modes of the H_4_nba ligand, see: Zhang *et al.* (2006 ▶). For related structures, see: Zhang *et al.* (2003 ▶).
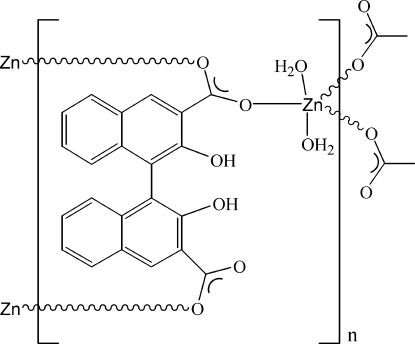


## Experimental

### 

#### Crystal data

[Zn(C_22_H_12_O_6_)(H_2_O)_2_]
*M* *_r_* = 473.72
Monoclinic, 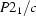

*a* = 15.4581 (19) Å
*b* = 9.5876 (10) Å
*c* = 13.4453 (14) Å
β = 90.047 (4)°
*V* = 1992.7 (4) Å^3^
*Z* = 4
Mo *K*α radiation
μ = 1.28 mm^−1^
*T* = 293 K
0.20 × 0.18 × 0.10 mm

#### Data collection

Bruker SMART APEX area-detector diffractometer
Absorption correction: multi-scan (*SADABS*; Bruker, 2001 ▶) *T*_min_ = 0.784, *T*_max_ = 0.883
10598 measured reflections
4323 independent reflections
3258 reflections with *I* > 2σ(*I*)
*R*_int_ = 0.032

#### Refinement

*R*[*F* ^2^ > 2σ(*F* ^2^)] = 0.045
*wR*(*F* ^2^) = 0.118
*S* = 1.06
4323 reflections
280 parameters
H-atom parameters constrained
Δρ_max_ = 0.42 e Å^−3^
Δρ_min_ = −0.35 e Å^−3^

### 

Data collection: *SMART* (Bruker, 2001 ▶); cell refinement: *SAINT* (Bruker, 2001 ▶); data reduction: *SAINT*; program(s) used to solve structure: *SHELXS97* (Sheldrick, 2008 ▶); program(s) used to refine structure: *SHELXL97* (Sheldrick, 2008 ▶); molecular graphics: *DIAMOND* (Brandenburg, 2006 ▶) and *OLEX* (Dolomanov *et al.*, 2003 ▶); software used to prepare material for publication: *SHELXTL* (Sheldrick, 2008 ▶).

## Supplementary Material

Crystal structure: contains datablocks I, global. DOI: 10.1107/S1600536809051836/ng2697sup1.cif

Structure factors: contains datablocks I. DOI: 10.1107/S1600536809051836/ng2697Isup2.hkl

Additional supplementary materials:  crystallographic information; 3D view; checkCIF report

## Figures and Tables

**Table 1:** Hydrogen-bond geometry (Å, °)

*D*—H⋯*A*	*D*—H	H⋯*A*	*D*⋯*A*	*D*—H⋯*A*
O2*W*—H2*WB*⋯O2	0.85	2.04	2.770 (3)	144
O2*W*—H2*WA*⋯O5^i^	0.85	1.95	2.719 (3)	150
O5—H5*A*⋯O2	0.82	1.77	2.517 (3)	150
O1*W*—H1*WB*⋯O6^ii^	0.85	2.19	2.863 (3)	136
O6—H6*A*⋯O3	0.82	1.89	2.607 (3)	146
O1*W*—H1*WA*⋯O2*W*^iii^	0.85	2.32	3.137 (3)	162

Symmetry codes: (i) 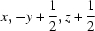; (ii) 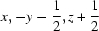; (iii) 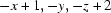.

**Fig. 1.**
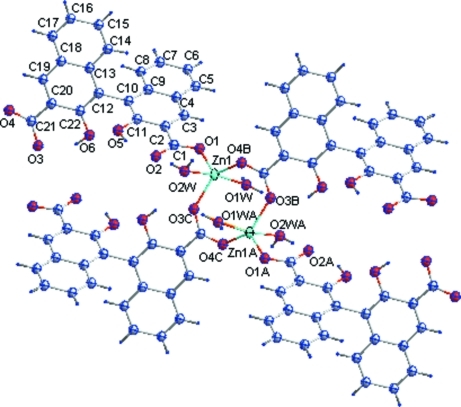
Part of the polymer network of the title complex, showing 50% probability displacement ellipsoids [Symmetry codes: (A) 1 - x, -y, 2 - z; (B) x, y, 1 + z; (C) 1 - x, -y, 1 - z]. Unlabelled atoms are related to labelled atoms by the sysmmetry operations (1 - x, -y, 2 - z; x, y, 1 + z; 1 - x, -y, 1 - z) (atoms C1–C22/O1–O6), respectively.

**Fig. 2.**
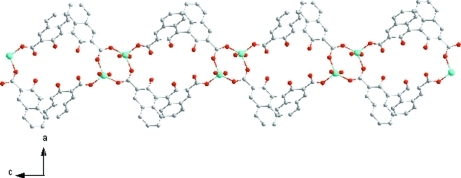
Perspective view showing the ZnII atoms connected by H2nba ligands into ribbons along the c axis. H atoms have been omitted for clarity.

**Fig. 3.**
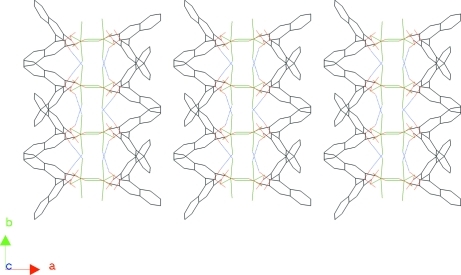
Packing diagram, viewed along the c axis. Dashed lines indicated hydrogen bonds. H atoms have been omitted for clarity.

## supplementary crystallographic information

### Comment

Recently much interest has been focused on the design and synthesis of coordination polymers with d^10^ metal ions and dicarboxylate not only for their interesting molecular topologies, but also for the fact that they may be designed with photoluminescence (Han *et al.* 2008; Zheng *et al.* 2004). The 2,2'-dihydroxy-[1,1']-binaphthalene-3,3'-dicarboxylic acid (H_4_nba) is a multifunctional ligand containing both carboxylic and hydroxy groups, which can potentially afford various coordination modes (Zhang *et al.*, 2006). Meanwhile, it also possesses both rigidity and flexibility, since the naphthyl rings can be twisted at some degrees across the C–C single bond due to steric effect. As an extension of our previous investigations, H_4_nba was introduced into zinc dicarboxylate system and the title coordination complex was isolated. In the title complex, each Zn^II^ atom is coordinated by five O atoms from three H_2_nba ligands and two aqua ligands in a distorted trigonal-bipyramidal geometry, with the two coordinated aqua ligands at the axial sites (Figure 1). Two Zn^II^ atoms related by a twofold axis are brideged by a pair of the H_2_nba ligands µ-carboxylate ends into a dinuclear unit [Zn1···Zn1A = 3.4971 (5) Å] and the distance is nearer than that of the *m*-phthalalate ligands (Zhang *et al.*, 2003). The H_2_nba ligands act in the mono bridging bidentate coordination mode to link the adjacent Zn^II^ atoms to form one-dimensional ribbons running along the *c* axis (Figure 2). These ribbons are assembled into layers paralled to the *bc* plane by the O–H···O hydrogen bonds. The O1w···O6^iv^ distance is 2.866 (3) Å and the O2w···O5^v^ distance is 2.715 (4) Å [symmetry codes: (iv) *x*, -1/2 - *y*, 1/2 + *z*; (v) *x*, 1/2 - *y*, 1/2 + *z*] (Figure 3 and Table 1).

### Experimental

A mixture of Zn(CH_3_COO)_2_ (0.184 g, 1 mmol), H_4_nba (0.094 g, 0.25 mmol) and NaOH (0.020 g, 0.50 mmol) in water (10 ml) was heated for 3 days at 130°C in a Parr Teflon-lined stainless steel vessel (23 ml), cooled to room temperature at a rate of 5 °C h^-1^. Pale-yellow crystals of the title complex were collected, washed with water and dried in air (yield 80%). IR data (KBr, cm^-1^): 3364*m*, 3057*m*, 1953w, 1832w, 1643 s, 1585*m*, 1505 s, 1457 s, 1397 s, 1340*m*, 1304*m*, 1239 s, 1152*m*, 1079w, 1007w, 939*m*, 866*m*, 807 s, 749 s, 624w, 598*m*, 440*m*. Anal. Calcd (%) for C_22_H_16_O_8_Zn: C, 55.78; H, 3.40. Found: C, 55.50; H, 3.72.

### Refinement

H atoms were positioned geometrically, with C—H = 0.95 (aromatic), 0.98(methyl), 0.99(methylene) and O—H = 0.82 Å, and refined as riding on their parent atoms with *U*_iso_(H)= 1.5*U*_eq_(C) for methyl H and 1.2*U*_eq_(C, O) for all other H.

### Crystal data

**Table:**

[Zn(C_22_H_12_O_6_)(H_2_O)_2_]	*F*(000) = 968
*M**_r_* = 473.72	*D*_x_ = 1.579 Mg m^−^^3^
Monoclinic, *P*2_1_/*c*	Mo *K*α radiation, λ = 0.71073 Å
Hall symbol: -P 2ybc	Cell parameters from 3258 reflections
*a* = 15.4581 (19) Å	θ = 2–27°
*b* = 9.5876 (10) Å	µ = 1.28 mm^−^^1^
*c* = 13.4453 (14) Å	*T* = 293 K
β = 90.047 (4)°	Block, yellow
*V* = 1992.7 (4) Å^3^	0.20 × 0.18 × 0.10 mm
*Z* = 4	

### Data collection

**Table:**

Bruker SMART APEX area-detector diffractometer	4323 independent reflections
Radiation source: fine-focus sealed tube	3258 reflections with *I* > 2σ(*I*)
graphite	*R*_int_ = 0.032
φ and ω scans	θ_max_ = 27.0°, θ_min_ = 2.5°
Absorption correction: multi-scan (*SADABS*; Bruker, 2001)	*h* = −19→9
*T*_min_ = 0.784, *T*_max_ = 0.883	*k* = −12→9
10598 measured reflections	*l* = −15→17

### Refinement

**Table:**

Refinement on *F*^2^	Primary atom site location: structure-invariant direct methods
Least-squares matrix: full	Secondary atom site location: difference Fourier map
*R*[*F*^2^ > 2σ(*F*^2^)] = 0.045	Hydrogen site location: inferred from neighbouring sites
*wR*(*F*^2^) = 0.118	H-atom parameters constrained
*S* = 1.06	*w* = 1/[σ^2^(*F*_o_^2^) + (0.0604*P*)^2^ + 0.1133*P*] where *P* = (*F*_o_^2^ + 2*F*_c_^2^)/3
4323 reflections	(Δ/σ)_max_ = 0.001
280 parameters	Δρ_max_ = 0.42 e Å^−^^3^
0 restraints	Δρ_min_ = −0.35 e Å^−^^3^

### Special details

**Table:**

Geometry. All e.s.d.'s (except the e.s.d. in the dihedral angle between two l.s. planes) are estimated using the full covariance matrix. The cell e.s.d.'s are taken into account individually in the estimation of e.s.d.'s in distances, angles and torsion angles; correlations between e.s.d.'s in cell parameters are only used when they are defined by crystal symmetry. An approximate (isotropic) treatment of cell e.s.d.'s is used for estimating e.s.d.'s involving l.s. planes.
Refinement. Refinement of *F*^2^ against ALL reflections. The weighted *R*-factor *wR* and goodness of fit *S* are based on *F*^2^, conventional *R*-factors *R* are based on *F*, with *F* set to zero for negative *F*^2^. The threshold expression of *F*^2^ > σ(*F*^2^) is used only for calculating *R*-factors(gt) *etc*. and is not relevant to the choice of reflections for refinement. *R*-factors based on *F*^2^ are statistically about twice as large as those based on *F*, and *R*- factors based on ALL data will be even larger.

### Fractional atomic coordinates and isotropic or equivalent isotropic displacement parameters (Å^2^)

**Table:**

	*x*	*y*	*z*	*U*_iso_*/*U*_eq_	
Zn1	0.57700 (2)	0.01105 (3)	0.90514 (2)	0.03018 (13)	
O1	0.64675 (16)	−0.0504 (2)	0.79378 (15)	0.0433 (6)	
O2	0.59198 (15)	0.1048 (2)	0.68741 (14)	0.0422 (6)	
O3	0.54356 (14)	−0.0097 (2)	0.14753 (16)	0.0420 (6)	
O4	0.63139 (15)	0.0708 (2)	0.02993 (14)	0.0395 (5)	
O5	0.64514 (15)	0.1116 (2)	0.51057 (14)	0.0408 (6)	
H5A	0.6186	0.1340	0.5609	0.061*	
O6	0.61219 (13)	−0.0839 (2)	0.31663 (14)	0.0339 (5)	
H6A	0.5752	−0.0822	0.2729	0.051*	
C1	0.6393 (2)	0.0014 (3)	0.7071 (2)	0.0326 (7)	
C2	0.69136 (19)	−0.0645 (3)	0.62605 (19)	0.0284 (6)	
C3	0.73883 (19)	−0.1821 (3)	0.6437 (2)	0.0307 (6)	
H3A	0.7375	−0.2221	0.7067	0.037*	
C4	0.78967 (18)	−0.2444 (3)	0.5691 (2)	0.0296 (6)	
C5	0.8370 (2)	−0.3682 (3)	0.5858 (2)	0.0374 (7)	
H5B	0.8364	−0.4084	0.6487	0.045*	
C6	0.8832 (2)	−0.4300 (3)	0.5123 (3)	0.0443 (8)	
H6B	0.9127	−0.5127	0.5246	0.053*	
C7	0.8861 (2)	−0.3681 (4)	0.4173 (3)	0.0488 (9)	
H7A	0.9177	−0.4104	0.3668	0.059*	
C8	0.8431 (2)	−0.2469 (3)	0.3988 (2)	0.0383 (7)	
H8A	0.8473	−0.2061	0.3362	0.046*	
C9	0.79223 (18)	−0.1818 (3)	0.47289 (19)	0.0285 (6)	
C10	0.74391 (18)	−0.0585 (3)	0.45474 (19)	0.0257 (6)	
C11	0.6935 (2)	−0.0044 (3)	0.5296 (2)	0.0280 (6)	
C12	0.74652 (19)	0.0145 (3)	0.35614 (19)	0.0253 (6)	
C13	0.81783 (19)	0.1036 (3)	0.3325 (2)	0.0294 (6)	
C14	0.8882 (2)	0.1224 (4)	0.3978 (2)	0.0453 (8)	
H14A	0.8891	0.0753	0.4583	0.054*	
C15	0.9553 (2)	0.2087 (4)	0.3735 (3)	0.0583 (10)	
H15A	1.0015	0.2191	0.4173	0.070*	
C16	0.9551 (2)	0.2816 (4)	0.2834 (3)	0.0589 (11)	
H16A	1.0005	0.3415	0.2682	0.071*	
C17	0.8890 (2)	0.2652 (4)	0.2181 (2)	0.0480 (9)	
H17A	0.8901	0.3129	0.1579	0.058*	
C18	0.81814 (19)	0.1762 (3)	0.2405 (2)	0.0312 (7)	
C19	0.7502 (2)	0.1528 (3)	0.1732 (2)	0.0304 (7)	
H19A	0.7516	0.1971	0.1117	0.036*	
C20	0.68185 (18)	0.0671 (3)	0.19490 (18)	0.0238 (6)	
C21	0.6144 (2)	0.0419 (3)	0.1182 (2)	0.0284 (6)	
C22	0.67968 (18)	−0.0008 (3)	0.28986 (19)	0.0236 (6)	
O1W	0.56864 (16)	−0.2006 (2)	0.95518 (16)	0.0462 (6)	
H1WA	0.5280	−0.2235	0.9944	0.069*	
H1WB	0.6059	−0.2594	0.9350	0.069*	
O2W	0.56514 (17)	0.2292 (2)	0.87072 (16)	0.0518 (7)	
H2WA	0.6061	0.2687	0.9023	0.078*	
H2WB	0.5809	0.2283	0.8102	0.078*	

### Atomic displacement parameters (Å^2^)

**Table:**

	*U*^11^	*U*^22^	*U*^33^	*U*^12^	*U*^13^	*U*^23^
Zn1	0.0311 (2)	0.0431 (2)	0.01629 (17)	−0.00210 (16)	−0.00209 (13)	−0.00114 (13)
O1	0.0522 (15)	0.0580 (13)	0.0198 (10)	0.0129 (12)	0.0082 (10)	0.0063 (10)
O2	0.0533 (15)	0.0479 (13)	0.0254 (11)	0.0149 (11)	0.0027 (10)	0.0015 (9)
O3	0.0338 (12)	0.0618 (15)	0.0303 (12)	−0.0129 (11)	−0.0120 (10)	0.0151 (10)
O4	0.0500 (14)	0.0508 (13)	0.0177 (10)	−0.0138 (11)	−0.0080 (9)	0.0024 (9)
O5	0.0615 (15)	0.0406 (12)	0.0202 (10)	0.0186 (11)	0.0043 (10)	0.0032 (9)
O6	0.0325 (11)	0.0461 (12)	0.0232 (10)	−0.0123 (10)	−0.0070 (9)	0.0098 (9)
C1	0.0366 (17)	0.0391 (17)	0.0221 (14)	0.0005 (14)	0.0012 (13)	0.0020 (12)
C2	0.0308 (15)	0.0349 (15)	0.0196 (13)	0.0001 (13)	−0.0016 (12)	0.0017 (11)
C3	0.0348 (16)	0.0376 (16)	0.0198 (13)	−0.0018 (14)	−0.0028 (12)	0.0074 (12)
C4	0.0263 (15)	0.0331 (15)	0.0293 (15)	−0.0024 (12)	−0.0048 (12)	0.0026 (12)
C5	0.0334 (17)	0.0378 (17)	0.0411 (18)	−0.0003 (14)	−0.0046 (14)	0.0100 (14)
C6	0.040 (2)	0.0373 (18)	0.055 (2)	0.0081 (16)	−0.0019 (17)	0.0038 (16)
C7	0.046 (2)	0.053 (2)	0.048 (2)	0.0129 (17)	0.0067 (17)	−0.0114 (16)
C8	0.0387 (18)	0.0488 (19)	0.0274 (16)	0.0058 (15)	−0.0008 (14)	−0.0037 (13)
C9	0.0259 (15)	0.0354 (16)	0.0241 (14)	−0.0025 (12)	−0.0057 (12)	−0.0002 (12)
C10	0.0286 (15)	0.0311 (14)	0.0174 (13)	−0.0010 (12)	−0.0056 (11)	0.0035 (11)
C11	0.0324 (15)	0.0315 (15)	0.0201 (13)	0.0029 (13)	−0.0030 (12)	0.0001 (11)
C12	0.0310 (15)	0.0287 (14)	0.0162 (12)	0.0006 (12)	−0.0008 (11)	0.0011 (11)
C13	0.0305 (15)	0.0348 (16)	0.0230 (14)	−0.0043 (13)	−0.0038 (12)	−0.0027 (12)
C14	0.041 (2)	0.063 (2)	0.0315 (17)	−0.0153 (17)	−0.0100 (15)	0.0038 (15)
C15	0.043 (2)	0.083 (3)	0.048 (2)	−0.026 (2)	−0.0156 (18)	−0.002 (2)
C16	0.042 (2)	0.077 (3)	0.059 (2)	−0.032 (2)	−0.0020 (19)	0.004 (2)
C17	0.050 (2)	0.057 (2)	0.0376 (18)	−0.0211 (18)	0.0020 (16)	0.0081 (15)
C18	0.0313 (16)	0.0357 (16)	0.0264 (14)	−0.0056 (13)	0.0012 (12)	−0.0003 (12)
C19	0.0388 (17)	0.0325 (15)	0.0198 (13)	−0.0013 (13)	−0.0003 (12)	0.0049 (11)
C20	0.0267 (14)	0.0269 (13)	0.0178 (12)	−0.0006 (12)	−0.0030 (11)	−0.0003 (11)
C21	0.0373 (17)	0.0278 (14)	0.0199 (13)	−0.0029 (13)	−0.0048 (12)	0.0023 (11)
C22	0.0287 (14)	0.0243 (13)	0.0179 (12)	−0.0006 (12)	0.0008 (11)	−0.0001 (10)
O1W	0.0625 (16)	0.0376 (12)	0.0384 (13)	0.0087 (11)	0.0125 (11)	0.0014 (10)
O2W	0.0822 (19)	0.0437 (13)	0.0294 (12)	−0.0184 (13)	−0.0089 (12)	0.0003 (10)

### Geometric parameters (Å, °)

**Table:**

Zn1—O1	1.937 (2)	C7—H7A	0.9300
Zn1—O4^i^	1.9616 (19)	C8—C9	1.415 (4)
Zn1—O3^ii^	1.993 (2)	C8—H8A	0.9300
Zn1—O1W	2.142 (2)	C9—C10	1.419 (4)
Zn1—O2W	2.150 (2)	C10—C11	1.375 (4)
O1—C1	1.273 (3)	C10—C12	1.500 (3)
O2—C1	1.259 (3)	C12—C22	1.372 (4)
O3—C21	1.265 (4)	C12—C13	1.431 (4)
O3—Zn1^ii^	1.993 (2)	C13—C14	1.410 (4)
O4—C21	1.247 (3)	C13—C18	1.420 (4)
O4—Zn1^iii^	1.9616 (19)	C14—C15	1.367 (5)
O5—C11	1.364 (3)	C14—H14A	0.9300
O5—H5A	0.8200	C15—C16	1.399 (5)
O6—C22	1.361 (3)	C15—H15A	0.9300
O6—H6A	0.8200	C16—C17	1.355 (5)
C1—C2	1.495 (4)	C16—H16A	0.9300
C2—C3	1.366 (4)	C17—C18	1.421 (4)
C2—C11	1.419 (4)	C17—H17A	0.9300
C3—C4	1.407 (4)	C18—C19	1.403 (4)
C3—H3A	0.9300	C19—C20	1.371 (4)
C4—C5	1.412 (4)	C19—H19A	0.9300
C4—C9	1.427 (4)	C20—C22	1.433 (3)
C5—C6	1.355 (4)	C20—C21	1.486 (4)
C5—H5B	0.9300	O1W—H1WA	0.8502
C6—C7	1.409 (5)	O1W—H1WB	0.8499
C6—H6B	0.9300	O2W—H2WA	0.8500
C7—C8	1.362 (5)	O2W—H2WB	0.8500
			
O1—Zn1—O4^i^	120.75 (10)	C9—C10—C12	121.7 (2)
O1—Zn1—O3^ii^	104.14 (10)	O5—C11—C10	118.8 (2)
O4^i^—Zn1—O3^ii^	134.89 (10)	O5—C11—C2	119.3 (2)
O1—Zn1—O1W	89.35 (9)	C10—C11—C2	122.0 (3)
O4^i^—Zn1—O1W	91.94 (9)	C22—C12—C13	120.0 (2)
O3^ii^—Zn1—O1W	92.80 (9)	C22—C12—C10	120.2 (2)
O1—Zn1—O2W	100.18 (9)	C13—C12—C10	119.8 (2)
O4^i^—Zn1—O2W	86.36 (9)	C14—C13—C18	118.5 (3)
O3^ii^—Zn1—O2W	81.39 (9)	C14—C13—C12	122.2 (3)
O1W—Zn1—O2W	169.80 (9)	C18—C13—C12	119.3 (3)
C1—O1—Zn1	122.6 (2)	C15—C14—C13	120.9 (3)
C21—O3—Zn1^ii^	134.52 (19)	C15—C14—H14A	119.5
C21—O4—Zn1^iii^	131.2 (2)	C13—C14—H14A	119.5
C11—O5—H5A	109.5	C14—C15—C16	120.5 (3)
C22—O6—H6A	109.5	C14—C15—H15A	119.7
O2—C1—O1	123.5 (3)	C16—C15—H15A	119.7
O2—C1—C2	119.5 (3)	C17—C16—C15	120.3 (3)
O1—C1—C2	117.0 (3)	C17—C16—H16A	119.9
C3—C2—C11	118.7 (3)	C15—C16—H16A	119.9
C3—C2—C1	120.8 (2)	C16—C17—C18	120.9 (3)
C11—C2—C1	120.5 (3)	C16—C17—H17A	119.6
C2—C3—C4	121.8 (3)	C18—C17—H17A	119.6
C2—C3—H3A	119.1	C19—C18—C13	118.7 (3)
C4—C3—H3A	119.1	C19—C18—C17	122.5 (3)
C3—C4—C5	122.2 (3)	C13—C18—C17	118.8 (3)
C3—C4—C9	118.9 (3)	C20—C19—C18	122.4 (2)
C5—C4—C9	118.9 (3)	C20—C19—H19A	118.8
C6—C5—C4	121.7 (3)	C18—C19—H19A	118.8
C6—C5—H5B	119.2	C19—C20—C22	118.7 (2)
C4—C5—H5B	119.2	C19—C20—C21	119.4 (2)
C5—C6—C7	119.6 (3)	C22—C20—C21	121.9 (2)
C5—C6—H6B	120.2	O4—C21—O3	124.5 (3)
C7—C6—H6B	120.2	O4—C21—C20	118.4 (3)
C8—C7—C6	120.6 (3)	O3—C21—C20	117.0 (2)
C8—C7—H7A	119.7	O6—C22—C12	117.9 (2)
C6—C7—H7A	119.7	O6—C22—C20	121.3 (2)
C7—C8—C9	121.2 (3)	C12—C22—C20	120.8 (2)
C7—C8—H8A	119.4	Zn1—O1W—H1WA	119.0
C9—C8—H8A	119.4	Zn1—O1W—H1WB	119.1
C8—C9—C10	122.6 (3)	H1WA—O1W—H1WB	121.9
C8—C9—C4	117.9 (3)	Zn1—O2W—H2WA	105.2
C10—C9—C4	119.4 (2)	Zn1—O2W—H2WB	99.9
C11—C10—C9	119.1 (2)	H2WA—O2W—H2WB	105.6
C11—C10—C12	119.1 (2)		
			
O4^i^—Zn1—O1—C1	−126.7 (2)	C9—C10—C12—C22	102.1 (3)
O3^ii^—Zn1—O1—C1	48.7 (3)	C11—C10—C12—C13	100.1 (3)
O1W—Zn1—O1—C1	141.4 (3)	C9—C10—C12—C13	−79.7 (3)
O2W—Zn1—O1—C1	−34.9 (3)	C22—C12—C13—C14	−179.7 (3)
Zn1—O1—C1—O2	6.6 (5)	C10—C12—C13—C14	2.1 (4)
Zn1—O1—C1—C2	−174.6 (2)	C22—C12—C13—C18	0.4 (4)
O2—C1—C2—C3	−175.7 (3)	C10—C12—C13—C18	−177.7 (3)
O1—C1—C2—C3	5.3 (4)	C18—C13—C14—C15	0.2 (5)
O2—C1—C2—C11	5.4 (4)	C12—C13—C14—C15	−179.7 (3)
O1—C1—C2—C11	−173.6 (3)	C13—C14—C15—C16	0.6 (6)
C11—C2—C3—C4	0.2 (4)	C14—C15—C16—C17	−1.3 (6)
C1—C2—C3—C4	−178.7 (3)	C15—C16—C17—C18	1.2 (6)
C2—C3—C4—C5	−178.1 (3)	C14—C13—C18—C19	177.0 (3)
C2—C3—C4—C9	0.7 (4)	C12—C13—C18—C19	−3.1 (4)
C3—C4—C5—C6	177.6 (3)	C14—C13—C18—C17	−0.3 (4)
C9—C4—C5—C6	−1.3 (5)	C12—C13—C18—C17	179.6 (3)
C4—C5—C6—C7	1.5 (5)	C16—C17—C18—C19	−177.6 (3)
C5—C6—C7—C8	0.1 (5)	C16—C17—C18—C13	−0.4 (5)
C6—C7—C8—C9	−2.0 (5)	C13—C18—C19—C20	2.8 (4)
C7—C8—C9—C10	−177.4 (3)	C17—C18—C19—C20	−180.0 (3)
C7—C8—C9—C4	2.2 (5)	C18—C19—C20—C22	0.2 (4)
C3—C4—C9—C8	−179.4 (3)	C18—C19—C20—C21	−177.1 (3)
C5—C4—C9—C8	−0.6 (4)	Zn1^iii^—O4—C21—O3	−17.4 (5)
C3—C4—C9—C10	0.2 (4)	Zn1^iii^—O4—C21—C20	161.5 (2)
C5—C4—C9—C10	179.0 (3)	Zn1^ii^—O3—C21—O4	−24.1 (5)
C8—C9—C10—C11	177.6 (3)	Zn1^ii^—O3—C21—C20	157.0 (2)
C4—C9—C10—C11	−1.9 (4)	C19—C20—C21—O4	17.1 (4)
C8—C9—C10—C12	−2.5 (4)	C22—C20—C21—O4	−160.1 (3)
C4—C9—C10—C12	177.9 (3)	C19—C20—C21—O3	−163.9 (3)
C9—C10—C11—O5	−178.0 (3)	C22—C20—C21—O3	18.9 (4)
C12—C10—C11—O5	2.2 (4)	C13—C12—C22—O6	−178.4 (2)
C9—C10—C11—C2	2.9 (4)	C10—C12—C22—O6	−0.2 (4)
C12—C10—C11—C2	−176.9 (3)	C13—C12—C22—C20	2.6 (4)
C3—C2—C11—O5	178.8 (3)	C10—C12—C22—C20	−179.2 (2)
C1—C2—C11—O5	−2.3 (4)	C19—C20—C22—O6	178.1 (2)
C3—C2—C11—C10	−2.0 (4)	C21—C20—C22—O6	−4.7 (4)
C1—C2—C11—C10	176.9 (3)	C19—C20—C22—C12	−3.0 (4)
C11—C10—C12—C22	−78.1 (3)	C21—C20—C22—C12	174.2 (3)

Symmetry codes: (i) *x*, *y*, *z*+1; (ii) −*x*+1, −*y*, −*z*+1; (iii) *x*, *y*, *z*−1.

### Hydrogen-bond geometry (Å, °)

**Table:**

*D*—H···*A*	*D*—H	H···*A*	*D*···*A*	*D*—H···*A*
O2W—H2WB···O2	0.85	2.04	2.770 (3)	144
O2W—H2WA···O5^iv^	0.85	1.95	2.719 (3)	150
O5—H5A···O2	0.82	1.77	2.517 (3)	150
O1W—H1WB···O6^v^	0.85	2.19	2.863 (3)	136
O6—H6A···O3	0.82	1.89	2.607 (3)	146
O1W—H1WA···O2W^vi^	0.85	2.32	3.137 (3)	162

Symmetry codes: (iv) *x*, −*y*+1/2, *z*+1/2; (v) *x*, −*y*−1/2, *z*+1/2; (vi) −*x*+1, −*y*, −*z*+2.
